# Genome-Wide Characterization and Expression Analysis of the Cysteine-Rich Polycomb-like Protein Gene Family in Response to Hormone Signaling in Apple (*Malus domestica*)

**DOI:** 10.3390/ijms26125528

**Published:** 2025-06-10

**Authors:** Le Jiang, Min Zhu, Ying Huang, Quanyan Zhang

**Affiliations:** 1 Shandong Provincial Key Laboratory of Water and Soil Conservation and Environmental Protection, College of Resources and Environment, Linyi University, Linyi 276000, China; jiangle@lyu.edu.cn; 2 College of Agriculture and Forestry Sciences, Linyi University, Linyi 276000, China; minzhu021030@163.com (M.Z.); huangying@lyu.edu.cn (Y.H.)

**Keywords:** apple, *CPP* gene family, expression profile, abiotic stresses, hormone

## Abstract

Cysteine-rich polycomb-like protein (CPP) transcription factors play critical roles in plant growth, development, and responses to stresses and hormone signaling. However, the research on the *CPP* gene family remains unexplored in apple. In this study, a total of 10 *CPP* genes (*MdCPP1*–*MdCPP10*) were identified and unevenly distributed across seven scaffolds. Phylogenetic and conserved motif analyses revealed that these 10 CXC domain-containing MdCPPs could be classified into three subfamilies. Evolutionary tree and synteny analyses demonstrated that apple shared the highest number of orthologous gene pairs with white pear compared to *Arabidopsis*. By analyzing the *MdCPP* gene promoter, a large number of *cis*-acting elements related to hormone and stress response were discovered. In addition, transcriptomic data demonstrated tissue-specific expression patterns of *MdCPP* genes, with *MdCPP5* and *MdCPP8* showing the highest expression in buds and leaves. The qRT-PCR results indicated that *MdCPP* genes have different expression responses to SA, GA, JA, and IAA treatments. Notably, *MdCPP4*, *MdCPP6*, *MdCPP8*, and *MdCPP9* were significantly upregulated under different hormone treatments. Among them, the upregulation of *MdCPP6* was the most significant. These findings establish a foundation for further functional characterization of *MdCPPs* and provide theoretical support for their potential applications in apple genetic improvement and agricultural production.

## 1. Introduction

Plants serve as the cornerstone of the global carbon sink system and are indispensable for human survival and development. However, with intensifying global climate change, the frequency and severity of constraints on plant growth and development are projected to escalate, posing substantial threats to the global carbon sink system [1,2,3,4]. To counteract these environmental challenges, plants have evolved a sophisticated set of regulatory mechanisms and signaling pathways that mediate the balance between growth, development, and stress defense, thereby optimizing survival efficiency [5,6,7].

Transcription factors (TFs), functioning as molecular switches for gene expression regulation, play pivotal roles in plant stress signaling by orchestrating the expression of multiple stress-responsive genes [8,9,10,11]. Transcription factors such as MYB [4], WRKY [12], NAC [13], and MYC [14] have been identified and characterized in plants. Among them, the cysteine-rich polycomb-like protein is one of the transcription factors widely distributed in plants and is almost ubiquitously present in flowering plants, both monocots and dicots [15,16]. CPP Transcription factors typically contain one or two CXC domains (PF03638), which are characterized by a conserved sequence motif (CXCX4CX3YCXCX6CX3CXCX2C), interspersed with a short R motif (RNPXAFXPK) [15]. Evolutionary studies suggested that there is coordinated co-evolution between the two CXC domains and their intervening R motif [17]. As a compact TF family, CPP can enhance stress resistance by regulating the development of plant structures, such as leaves and floral tissues, thereby helping plants adapt to extreme environments [18]. For instance, *AtCPP4* and *AtCPP5* play an important regulatory role in the differentiation and formation in Arabidopsis [19]. In tomato, all *CPP* genes, except SlCPP5, exhibit drought-induced upregulation compared to the control [20]. Salt stress triggered pronounced expression changes of *CPP* genes in Brassica napus, with *BoCPP1* and *BoCPP4* showing remarkable 140-fold and 80-fold induction, respectively [21]. In potato, hormonal response analyses reveal methyl jasmonate (MeJA)-mediated upregulation of StCPP-3 [22]. In addition, wheat *TaCPP3-1B* demonstrates consistently elevated transcript levels at all timepoints following exogenous indole-3-acetic acid (IAA) and 1-aminocyclopropane-1-carboxylic acid (ACC) treatments [23].

Apple (*Malus domestica*), a perennial deciduous fruit tree, has been the subject of some research on growth regulation and stress response, benefiting from advancements in functional genomics; however, no systematic investigation of *MdCPPs* has been reported to date. This study systematically identified 10 *MdCPP* family members using the apple whole-genome dataset (Version 1.1). Genome-wide analysis was conducted to investigate the physicochemical properties, chromosomal localization, gene structures, and intra- and interspecific synteny relationships of *MdCPPs*. Furthermore, the expression characteristics of *MdCPPs* were analyzed across different tissues, and qRT-PCR was employed to determine the expression patterns under different hormone treatments, revealing the diverse regulatory roles of *MdCPPs*. Additionally, *cis*-acting elements and protein interaction networks of MdCPPs were predicted. This research lays a theoretical foundation for further functional exploration of the *CPP* gene family in apple.

## 2. Results

### 2.1. Genome-Wide Identification and Phylogenetic Analysis of the CPP in Apple

**Figure 1:**
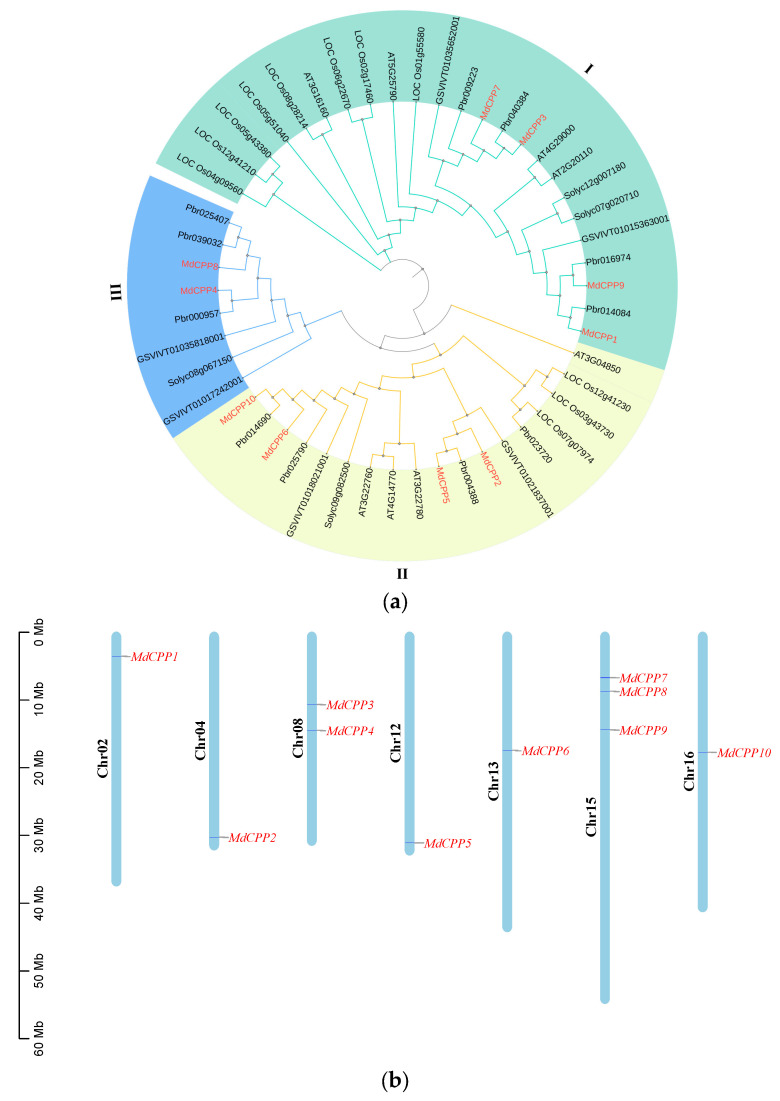
Phylogenetic tree and chromosomal localization of the CPPs in apple. (**a**) Phylogenetic analysis of CPPs from apple, *Arabidopsis*, rice, tomato, pear, and grape. Different colors represent distinct subfamilies. Genes highlighted in red indicate *MdCPPs*. (**b**) Distribution of *CPPs* on apple chromosomes. Chromosome numbers are labeled on the left side of each chromosome, and the names of *MdCPPs* are shown on the right. The distance in megabases (Mb) between genes or from the gene to the end of the chromosome has been given at the start of chromosomes.

Using a hidden Markov model (HMM), apple genes encoding proteins with the PF03638 domain were screened (Figure S1). By aligning eight known Arabidopsis CPP protein sequences from PlantTFDB [24] with apple protein data, a total of 10 MdCPPs (named MdCPP1 to MdCPP10) were identified in apple (Figure 1a; Table S1) Furthermore, chromosomal localization analysis revealed that MdCPPs are distributed across seven scaffolds. The results showed that *MdCPP7*, *MdCPP8*, and *MdCPP9* were located on scaffold 15, and *MdCPP3* and *MdCPP4* were located on scaffold 8, while scaffolds 2, 4, 12, 13, and 16 each contained a single gene.

To characterize the physicochemical properties of CPP proteins in apple, TBtools-II [25] was used for analysis. As shown in Table S2, the protein lengths of MdCPPs ranged from 455 amino acids (MdCPP2) to 900 amino acids (MdCPP5), with molecular weights varying between 49,404.47 Da (MdCPP2) and 97,609.08 Da (MdCPP5). The isoelectric points (pI) ranged from 5.38 (MdCPP1) to 9.05 (MdCPP2), the instability indices ranged from 50.9 (MdCPP1) to 71.96 (MdCPP2), and the aliphatic indices ranged from 59.54 (MdCPP7) to 73.54 (MdCPP2). Meanwhile, all the CPP proteins exhibited hydrophilic properties, with a grand average of hydropathicity values ranging from −0.754 (MdCPP8) to −0.438 (MdCPP2). According to Cell-PLoc 2.0 [26] prediction, all the MdCPP proteins were localized in the nucleus (Table S2).

In order to investigate evolutionary relationships, a phylogenetic tree was constructed. Based on topological clustering of the evolutionary tree, the CPPs were divided into three subfamilies (I–III), each with a distribution of CPP family members (Figure 1a), suggesting that CPPs may have gained functional diversity during differentiation. Notably, CPP proteins from apple, pear, grape, tomato, and Arabidopsis displayed intertwined evolutionary relationships rather than species-specific clustering, indicating that the *CPP* genes had undergone significant expansion. In addition, multiple MdCPPs clustered closely with PbrCPPs. For instance, MdCPP4 and Pbr000957 were located in Clade III. MdCPP1 and Pbr014084, MdCPP8 and Pbr016974, and MdCPP3 with Pbr040384, belonged to Clade I. Meanwhile, MdCPP5 and Pbr004388, as well as MdCPP10 and Pbr014690, were found in Clade II (Figure 1a). Hence, these phylogenetic patterns in CPPs showed high sequence homology and parallel evolutionary trends between apple and pear, implying that they may have conserved biological functions.

### 2.2. Multiple Sequence Alignment and Gene Structure Analysis of MdCPPs

**Figure 2:**
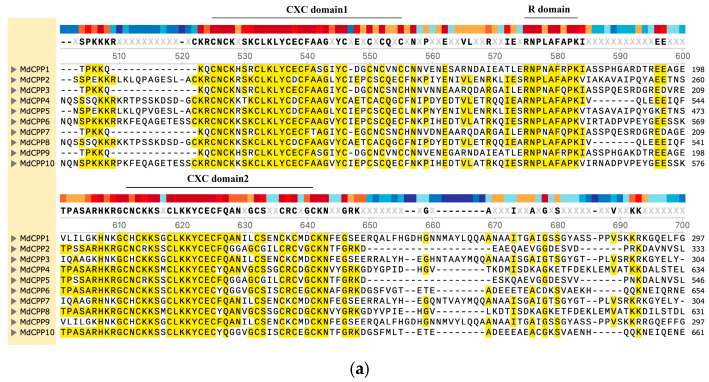

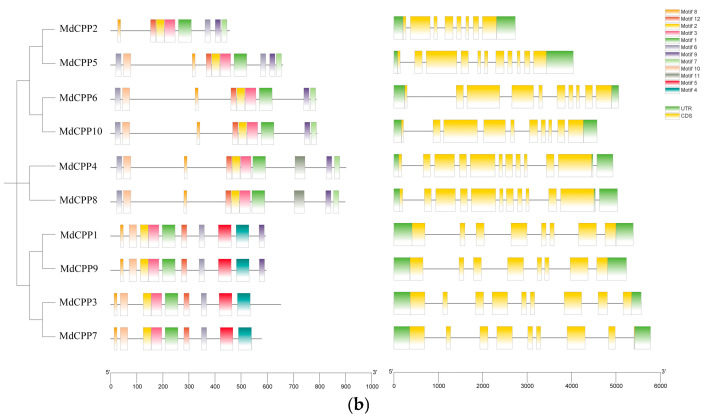
Sequence alignment and gene structure of MdCPPs. (**a**) Multiple sequence alignment of the CXC domains in MdCPP proteins. Different colors represent varying degrees of sequence conservation. (**b**) Conserved motifs and exon–intron structures of MdCPPs. Green boxes represent untranslated regions (UTRs), and yellow boxes indicate coding sequences (CDSs).

The CXC domain is a critical region for the biological functions of CPP proteins. Sequence alignment of MdCPP proteins revealed that all members contain two conserved CXC domains and an R motif connecting them (Figure 2a). To identify the conserved motifs in CPPs, MEME was employed to analyze 12 conserved motifs in apple (Figure 2b). The results revealed variations in motif distribution among MdCPPs. Each of the 10 MdCPP proteins contained 8 to 10 conserved motifs, with motifs 1, 2, 3, 8, and 12 present in all members. Notably, motifs 1, 2, and 3 were identified as CRC domains. Certain motifs exhibited subfamily-specific conservation; for instance, MdCPP4 and MdCPP8 shared identical conserved motifs.

Moreover, the exon–intron compositions of gene families often reflect evolutionary information of the gene family. The results of gene structure analysis showed that MdCPPs contained 8 to 11 exons and 7 to 10 introns, with subfamily members displaying similar exon–intron numbers (Figure 2b). The genes with the most exons and introns were MdCPP4, MdCPP5, and MdCPP8, all of which contained 11 exons and 10 introns, respectively, while MdCPP1, MdCPP2, and MdCPP7 contained only 8 exons and 7 introns (Figure 2b). According to the results, consistent exon–intron numbers within subfamilies, aligned using conserved motif analysis, indicated relatively conserved gene structures in the MdCPP gene subfamilies.

### 2.3. Synteny Analysis of MdCPPs

**Figure 3:**
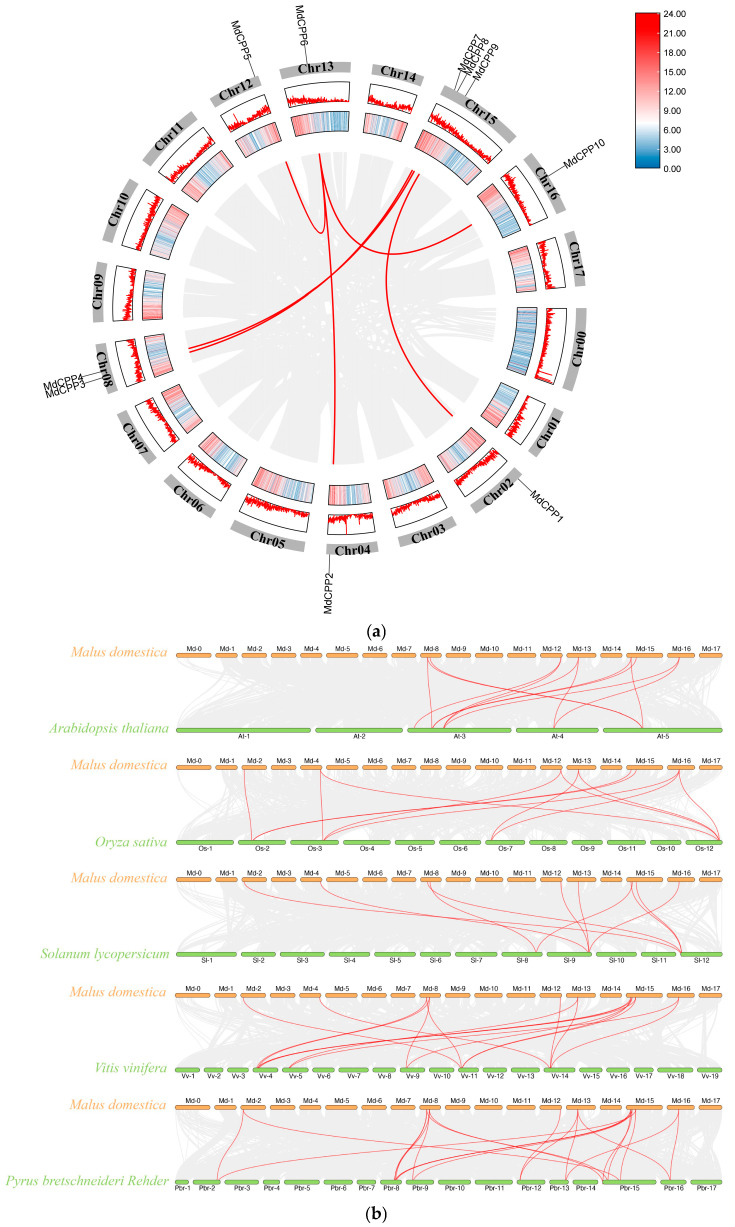
Synteny analysis of the *CPP* gene family. (**a**) Intra-species synteny analysis of *MdCPPs*. Red lines indicate duplication events of *MdCPPs*, and chromosome numbers are labeled within gray rectangles. The legend shows different colors corresponding to the number of genes in each chromosomal region, displayed as a heatmap and lines in the Circos plot. (**b**) Synteny analysis of *CPP* genes between apple and *Arabidopsis*, tomato, rice, grape, and pear. Gray lines in the background represent syntenic blocks between apple and other species, while homologous *CPP* gene pairs between species are highlighted by red lines.

Synteny analysis of the *CPP* gene family within apple was performed via TBtools, identifying six segmental duplication pairs among the 10 *MdCPPs*, such as *MdCPP1*/*MdCPP9*, *MdCPP2*/*MdCPP6*, *MdCPP3*/*MdCPP7*, *MdCPP4*/*MdCPP8*, *MdCPP5*/*MdCPP6*, and *MdCPP6*/*MdCPP10* (Figure 3a). These findings suggested that members within the same subfamily exhibited collinear relationships, and segmental duplication events played a pivotal role in the expansion of the *MdCPP* gene family.

To explore the evolutionary relationships of *CPP* members across different species, comparative synteny maps were constructed between *MdCPPs* and their homologs in *Arabidopsis*, pear, grape, tomato, and rice. The results revealed that *MdCPPs* exhibited the highest number of collinear relationships with pear, totaling 24 pairs, while they showed 17, 12, 12, and 10 pairs of collinear relationships with grape, *Arabidopsis*, rice, and tomato, respectively (Figure 3b). Notably, *MdCPPs* displayed the highest homology with pear, a perennial Rosaceae species, indicating shared evolutionary trajectories.

To assess evolutionary pressures on *MdCPPs*, the nonsynonymous (Ka) and synonymous (Ks) substitution rates were calculated for six homologous gene pairs. As shown in Supplementary Table S3, the Ka values were substantially lower than the Ks values across all pairs, resulting in Ka/Ks ratios of <1. This indicated that purifying selection had predominantly shaped the evolution of *MdCPPs*.

### 2.4. Secondary and Tertiary Structure Prediction of Apple CPP Proteins

**Figure 4:**
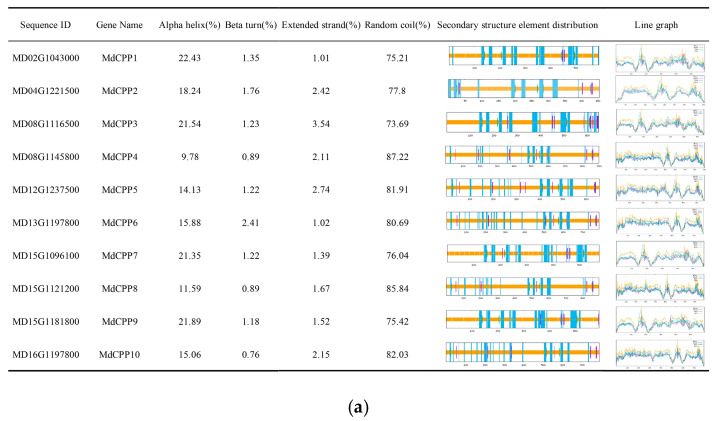

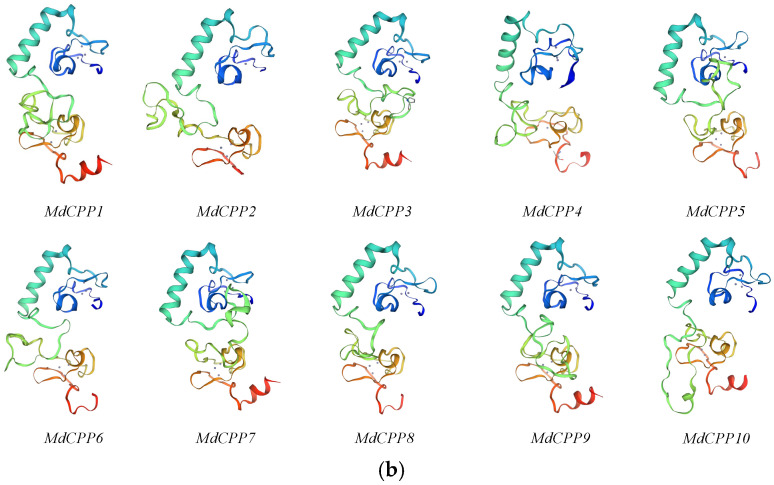
Prediction of secondary and tertiary structures of MdCPPs. (**a**) Diagram of the secondary structures of MdCPPs. Color assignments for secondary structure elements: α-Helix (blue), β-Sheet (purple), β-Turn (green), and Random Coil (yellow). (**b**) Tertiary structure models of MdCPPs.

Secondary structure prediction using the SOPMA online website revealed that MdCPP proteins were primarily composed of alpha-helices, beta-turns, extended strands, and random coils (Figure 4a). Among them, alpha-helices accounted for 9.78–22.43% of the structures, with MdCPP1 exhibiting the highest proportion while MdCPP4 showed the lowest. The proportion of *β*-turns and extended chains was relatively low. In addition, random coils accounted for the highest proportion, ranging from 73.69% to 87.22%. Thus, the secondary structure of MdCPP proteins was mainly composed of α-helices and random coils, indicating that CPP proteins may have unique advantages in maintaining structural stability and functional diversity.

The tertiary structure prediction using Expasy showed that all MdCPP proteins shared broadly similar architectures, predominantly composed of alpha-helices and random coils, consistent with the analysis results of the secondary structure predictions (Figure 4b). However, subtle structural variations among MdCPPs were observed, potentially linked to functional diversification within the gene family.

### 2.5. Cis-Acting Element Analysis of MdCPPs

**Figure 5:**
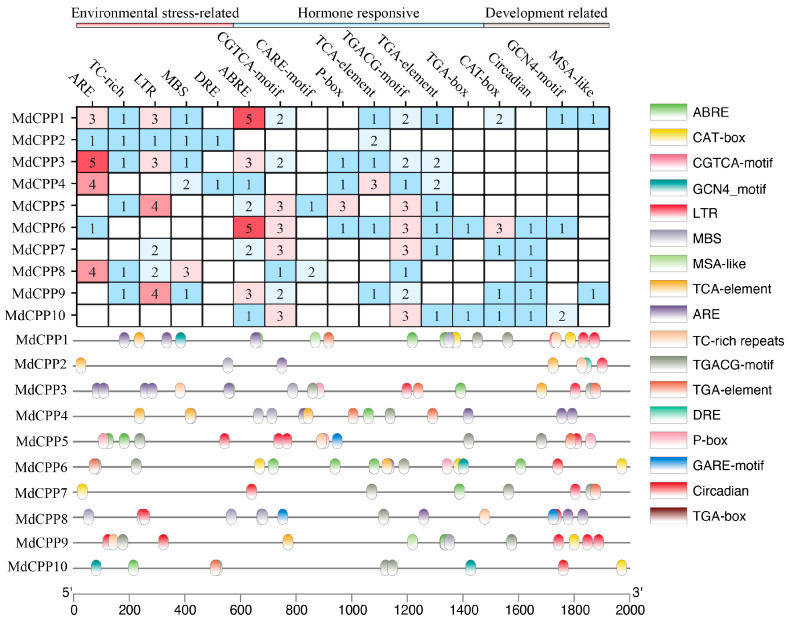
*Cis*-acting elements in the promoter regions of *MdCPPs*. The upper section shows the number and classification of various *cis*-acting elements in the promoter regions of each *MdCPP*. The lower section uses differently colored ovals to represent distinct types of *cis*-acting elements and their positions within the promoter regions.

*Cis*-acting elements in promoter regions play critical roles in regulating gene expression, and searching for conserved *cis*-acting elements can be used to predict gene functions. In order to further investigate the regulatory mechanisms of *MdCPPs*, *cis*-acting elements within the 2000 bp upstream regions of *MdCPP* coding sequences were analyzed using PlantCare (Figure 5). Three major categories of elements were identified. The first category included environmental stress-related elements such as low-temperature-responsive elements and drought-responsive elements. The second category comprised hormone-related elements, including auxin-responsive elements, gibberellin-responsive elements, abscisic acid-responsive elements, and salicylic acid-responsive elements. The third category comprised growth and development-related elements, such as endosperm development expression and meristematic tissue expression. Significantly, hormone and stress-responsive elements were the most abundant among the *MdCPPs*. In addition, *MdCPP1* contained the highest number of *cis*-acting elements, with a total of 23. Among all the genes, *MdCPP6* exhibited the most hormone-related elements, suggesting its potentially strong induction by hormonal treatments. Hormone-associated motifs such as ABRE, P-box, and CGTCA-motif were ubiquitous across all 10 *MdCPPs*, highlighting their regulatory roles in hormone signaling. Additionally, stress-responsive elements, including ARE, TC-rich repeats, LTR, MBS, and DRE, were identified, which may modulate *MdCPP* expression during plant stress responses.

### 2.6. Expression Pattern of MdCPPs

**Figure 6:**
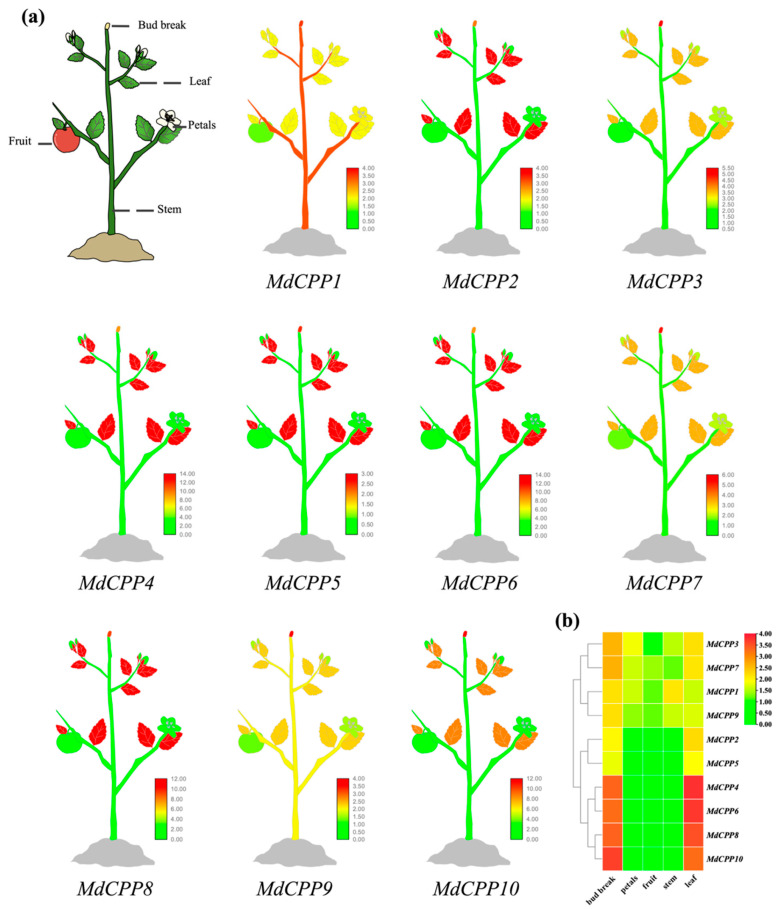
Expression analysis of *MdCPPs* in different tissues based on RNA-seq. (**a**) The first image provides annotations of different plant tissues, while the remaining 10 images represent cartoon heatmaps of gene expression in various tissues. Red indicates high gene expression levels, and green indicates low gene expression levels. (**b**) The heatmap was generated using mean values and plotted by TBtools. Red represents high expression levels, and green represents low expression levels. Expression levels were displayed using log2 (FPKM + 1) transformation.

To explore the potential functions of *MdCPPs* in apple development and stress responses, the expression patterns of CPP genes in different tissues and developmental stages were obtained and visualized as a plant tissue heatmap (Figure 6a). Most *MdCPPs* showed the highest expression levels during bud and leaf development, suggesting that MdCPPs may mainly play a role in plant growth and development stages and resistance to external environmental stress. Notably, the syntenic gene pairs *MdCPP2*/*MdCPP6*, *MdCPP3*/*MdCPP7*, *MdCPP4*/*MdCPP8*, and *MdCPP5*/*MdCPP6* displayed similar expression profiles across tissues. Previous studies have shown that *TSO1* mutants can regulate the development of buds and reproductive organs, and *MdCPP2*, a homologous gene, was highly expressed in leaves and buds (Figure 6b), suggesting that there may be functional conservation between *TSO1* and *MdCPP2* in developmental regulation.

### 2.7. Expression Profiling of MdCPPs Under Phytohormone Treatments Using qRT-PCR

**Figure 7:**
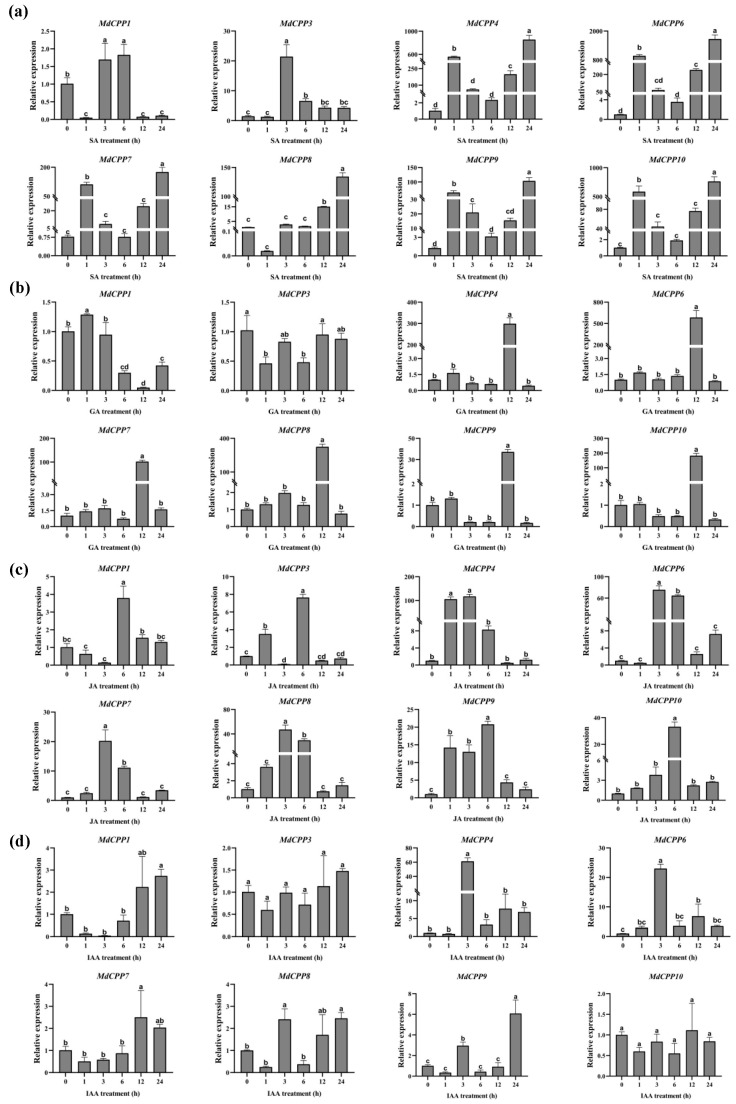
The qRT-PCR analysis of *MdCPPs* under hormone treatments. The control treatment refers to cultivating apple seedlings in nutrient solution, while the SA stress treatment refers to apple seedlings in nutrient solution with 100 μM SA added. Subsequently, apple seedlings were treated with 100 μM GA_3_, 20 μM JA, or 20 μM IAA in nutrient solution for the respective hormone treatments. Samples containing three biological replicates were collected at 0, 1, 3, 6, 12, and 24 h post-treatment, respectively. The x-axis represents the duration of hormone treatment (0, 1, 3, 6, 12, and 24 h), and the y-axis indicates the relative expression levels of genes. (**a**) Relative expression levels of *MdCPPs* in apple seedlings treated with 100 μM SA at 0, 1, 3, 6, 12, and 24 h. (**b**) Relative expression levels of *MdCPPs* in apple seedlings treated with 100 μM GA_3_ at 0, 1, 3, 6, 12, and 24 h. (**c**) Relative expression levels of *MdCPPs* in apple seedlings treated with 20 μM MeJA at 0, 1, 3, 6, 12, and 24 h. (**d**) Relative expression levels of *MdCPPs* in apple seedlings treated with 20 μM IAA at 0, 1, 3, 6, 12, and 24 h. Error bars represent standard deviation, and one-way ANOVA was performed. Different lower-case letters (e.g., a, b, c, d) denote statistically significant differences between groups (*p* < 0.05). Groups sharing the same letter are not significantly different. All experiments were performed independently at least three times.

To further investigate the potential roles of *MdCPPs* in hormone signaling pathways, this study analyzed the expression patterns of eight randomly selected *MdCPPs* under different phytohormone treatments using qRT-PCR. Apple seedlings were subjected to salicylic acid (SA), gibberellic acid (GA), jasmonic acid (JA), and indole-3-acetic acid (IAA) treatments, revealing distinct expression profiles among *MdCPPs* under various hormone treatments. Following SA treatment, the expression level of *MdCPP1* was significantly suppressed at 12 h post-treatment. Except for *MdCPP1*, the remaining *MdCPPs* exhibited similar expression patterns with significantly upregulated relative expression levels after treatment (Figure 7a). It is worth noting that the expression level of *MdCPP6* displayed the most dramatic response, showing a 1,671-fold increase in relative expression at 24 h compared to 0 h. Under GA treatment, *MdCPP4*, *MdCPP6*, *MdCPP7*, *MdCPP8*, *MdCPP9*, and *MdCPP10* reached peak expression levels at 12 h, while *MdCPP1* and *MdCPP3* demonstrated overall downregulation. The expression level of *MdCPP3* was suppressed throughout all the measured time points (Figure 7b). Meanwhile, JA treatment induced substantial upregulation of *MdCPP4*, *MdCPP6*, *MdCPP7*, *MdCPP8*, *MdCPP9*, and *MdCPP10* at 3 h and 6 h, displaying synchronized expression patterns (Figure 7c). Although partial suppression occurred at 12 h, most *MdCPPs* exhibited significant induction overall, particularly *MdCPP4,* with a hundred-fold increase. In addition, the expression levels of most genes were inhibited at 1 h after IAA treatment, such as *MdCPP1*, *MdCPP3*, *MdCPP7*, *MdCPP8*, and *MdCPP9*. In contrast, subfamily III member *MdCPP4* showed elevated expression at 24 h. Among the four hormone treatments, *MdCPP6* demonstrated the most pronounced upregulation, suggesting its pivotal role in hormone stress responses. These diverse expression patterns implied complex regulatory mechanisms of *MdCPPs* in hormone signaling.

### 2.8. Protein–Protein Interaction Analysis of MdCPPs

**Figure 8:**
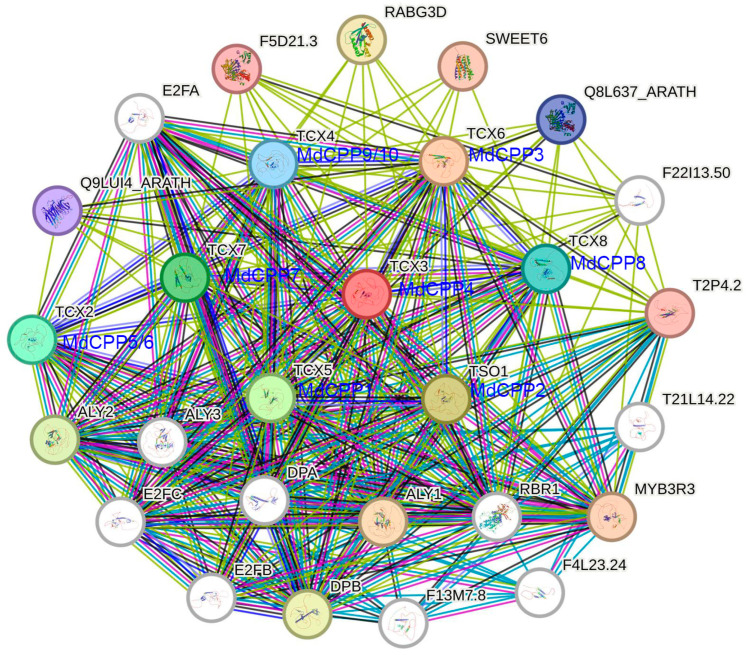
Protein–protein interaction analysis. Different line colors represent different types of protein–protein interactions. Colored nodes represent query proteins and first shell of interactors. White nodes represent second shell of interactors.

Protein interaction network prediction based on known interactomes represents an effective approach for investigating unknown protein networks in plants [27]. To further investigate the regulatory mechanisms of MdCPPs in growth, development, and response to hormone signals, STRING was used to predict their protein interaction network. The analysis revealed potential interactions between MdCPP proteins and MYB3R3, DPB, RABG3D, and other factors (Figure 8). MYB3R3 is a transcription repressor that regulates organ growth, which has been implicated in cold, salt, and drought stress responses [28]. Moreover, the interaction between DPB and SKP2A is regulated by auxin, while RABG3D is involved in intracellular vesicle trafficking and protein transport [29]. DPB exhibits co-expression relationships with all MdCPP proteins, suggesting that the expression of apple CPPs may be regulated by auxin. MdCPP proteins demonstrated complex interactions with various functional partners. For instance, MdCPP1, MdCPP3, MdCPP4, MdCPP7, MdCPP8, MdCPP9, and MdCPP10 interacted with Q8L637_ARATH and F5D21.3, which are associated with energy metabolism and cellular signaling. Co-expression relationships were observed between MdCPPs and others, indicating potential mutual regulatory mechanisms among these proteins.

## 3. Discussion

### 3.1. Identification and Characterization of CPPs in Apple

In response to environmental stresses, plants utilize transcription factors to regulate physiological processes, thereby enhancing their adaptability and stress resistance [30,31,32]. The CPP proteins are widely distributed across plants and animals, playing regulatory roles in the development of reproductive organs and controlling cell division in plants [15]. In this study, we conducted a genome-wide identification of the *MdCPP* gene family and investigated its functional implications during growth and development as well as hormone responses.

To date, CPPs have been identified in various species such as *Arabidopsis* [17], rice [17], tomato [20], soybean [33], and maize [34]. However, little is known about the identification and roles of CPPs in perennial woody plant trees, especially in apple. This study identified 10 *MdCPP* gene family members unevenly distributed across seven apple chromosomes (Figure 1b), suggesting the evolutionary divergence of *CPP* genes among species. Phylogenetic analysis incorporating CPP proteins from five dicotyledonous plants and one monocotyledonous plant was combined, and all the CPP proteins were divided into three branches according to the phylogenetic relationships (Figure 1a), consistent with the subfamily organization reported in bamboo [35]. Among these, each clade contained both monocotyledonous and dicotyledonous members, indicating that the CPP proteins originated prior to the divergence of monocots and dicots. Notably, no *Arabidopsis* members were detected in subfamily III, which was consistent with previous observations in bamboo, implying lineage-specific loss during evolution [35]. From the evolutionary relationships, members within the same clade, such as MdCPP1, MdCPP3, MdCPP7, and MdCPP9, exhibited highly conserved motifs and structures (Figure 2), suggesting that CPPs within the same branch may have similar functions.

A gene family is formed by the duplication of a single gene into two or more genes. Gene families typically expand through duplication events, which drive genome evolution [36,37,38,39,40]. Among the 10 members of the apple *CPP* gene family, 6 segmental duplication pairs were identified (Figure 3a), highlighting the critical role of segmental duplications in *MdCPP* gene family expansion. Interspecific collinearity analysis of CPP proteins from six species revealed 10 collinear relationships between *MdCPPs* and multiple tomato *CPP* genes (Figure 3b), indicating that these orthologous *CPP* genes were relatively conserved. Apple exhibited more collinear relationships with four other species compared to its own gene count, with 12 collinear pairs measured between seven *MdCPPs* and *Arabidopsis* genes, suggesting that multiple post-divergence duplication events contributed to family expansion [38]. Notably, apple displayed higher ortholog numbers with pear than with *Arabidopsis* (Figure 3b), mirroring the ortholog ratios observed between bamboo and rice or *Arabidopsis* [35]. This pattern may reflect divergent evolution between herbaceous and woody plants. The stronger collinearity between apple and other woody species could be linked to environmental adaptations in their respective habitats [27]. These findings collectively support species-specific expansion of the *CPP* gene family, a phenomenon corroborated in other gene families [41,42].

### 3.2. Roles of CPPs in Apple Development

Analysis of the tissue-specific expression patterns of the *MdCPP* gene family revealed that segmentally duplicated *MdCPPs* exhibit similar expression profiles. For example, *MdCPP6* and *MdCPP10* displayed comparable expression patterns during apple growth and development, enriched with *cis*-acting elements associated with plant development, such as CAT-box and TGA-box. Both genes showed high expression levels in buds, leaves, and fruits (Figure 6a). Previous studies have suggested that genes within the same subclade share similar sequence motifs and may perform analogous functions. For instance, *AtCPP4* and *AtCPP5*, both grouped in Clade A3, are known to regulate floral tissue development [43,44]. A similar trend was observed in *MdCPPs*, where members of Clade I (*MdCPP1*, *MdCPP3*, *MdCPP7*, and *MdCPP9*) exhibited consistently high expression levels during various growth stages, indicating their critical roles in apple development and functional conservation during evolution (Figure 6b). Transcriptomic data of *MdCPPs* in different tissues of apple revealed that *MdCPPs* were highly expressed in apple buds, particularly *MdCPP2*, which shared high similarity with *TSO1*, suggesting they may have similar functional characteristics in regulating bud and reproductive organ development [43,44]. Buds and leaves are two critical organs functioning in monitoring plant growth and adapting to stress. Healthy buds rapidly adapt growth strategies to environmental stresses, and their growth status is also related to the reproductive ability of plants [45,46,47,48]. Moreover, leaves drive the efficiency of photosynthesis, and healthy leaves can efficiently convert light energy and provide sufficient nutrients, enhancing stress resistance to maintain normal physiological functions [49,50,51]. The study revealed that *MdCPPs* were highly expressed in buds and leaves, suggesting their involvement in regulating organ development and stress adaptation, potentially through mechanisms such as cell proliferation, stress signaling, or transcriptional regulation. Consistently, CPP genes also exhibit bud-specific expression in wheat [23].

### 3.3. Response to Hormone Signaling of CPPs in Apple

Phytohormone signaling is crucial for plant growth, development, and abiotic stress responses. Subsequent qRT-PCR analysis was performed to validate the response characteristics of apple *CPP* members under various hormone treatments. Previous studies have shown that GA drives seed germination and stem elongation [52,53]. In this study, the collinear gene pairs *MdCPP4*/*MdCPP8* and *MdCPP6*/*MdCPP10* peaked at 12 h under GA treatment (Figure 7b), with reduced expression at 3 h and 6 h. The results indicated that genes from gene replication had similar functions and were relatively conserved in the evolutionary process. However, differentially expressed genes were also observed among these collinear gene pairs, such as *MdCPP3* and *MdCPP7*, indicating that long-term evolutionary processes have led to functional diversification of genes.

After SA treatment, the relative expression levels of all *MdCPPs* were upregulated to varying degrees (Figure 7a), likely mediated by SA-responsive *cis*-elements (e.g., TCA-element) in their promoters. As a key signaling molecule in plant immunity, SA enhances resistance to pathogen stress [54,55]. Previous studies have shown that the *MdVQ37-MdWRKY100* module regulates SA levels to combat *Glomerella leaf spot* in apple [56]. In this study, SA-induced *MdCPP* expression implied a potential role in disease resistance, providing new candidate genes for apple disease resistance breeding.

Similar to SA, JA is also believed to play a role in influencing plant resistance to pathogens and other stress factors [57]. As shown in Figure 5, promoter analysis showed that *MdCPPs* harbored JA-responsive motifs (TGACG-motif, CGTCA-motif). Meanwhile, expression analysis also showed that all *MdCPPs* were significantly induced at 6 h under JA treatment (Figure 7c), indicating that *MdCPPs* may mediate the JA process in response to pathogens and stress. Notably, the analysis also revealed *TCX8*, a known suppressor of JA biosynthesis that delays senescence [58]. In this study, *MdCPP8*, a homologous gene of *TCX8*, was significantly upregulated under JA treatment, suggesting its involvement in JA signaling. Studies have shown that SA and JA interact at multiple levels (crosstalk) and can jointly induce the expression of genes related to plant growth and defense [59,60]. In potato, *StCPP3* was upregulated upon MeJA treatment but downregulated by SA. Intriguingly, *StCPP3* suppressed the SA signaling pathway in response to Ralstonia solanacearum infection, suggesting a potential antagonistic interaction between these two hormones that may compromise plant disease resistance [22]. This study found that the application of the exogenous hormones SA and JA to apple seedlings promoted the expression of *MdCPPs*, suggesting that *MdCPPs* can respond to the induction of SA and JA and participate in the emergency regulation or defense response of apple seedlings (Figure 7a,c). In contrast, *MdCPPs* appear to exert synergistic effects through SA and JA pathway modulation, suggesting evolutionary divergence between woody and herbaceous species. Notably, the overexpression of *StCPP3* inhibited SA signaling, providing critical insights for our follow-up experimental design. It is worth noting that *MdCPP4*, *MdCPP6*, *MdCPP8*, and *MdCPP10* showed pronounced upregulation under SA, GA, and JA, indicating that *MdCPPs* strongly respond to these three hormones and may have a synergistic effect by regulating the signaling pathways of hormones [61,62]. This regulation could influence plant life processes, as demonstrated by the ability of SA to enhance rice disease resistance through the suppression of GA signaling or the reduction of GA levels [60]. However, hormone treatment experiments using fixed concentrations have certain limitations. Subsequent dose gradient experiments will be conducted to further elucidate the expression patterns of *MdCPPs*.

In summary, the genome-wide analysis of *MdCPPs* in this study may provide insights into their roles in plant growth, development, and hormone responses. Moreover, the findings offer valuable implications for molecular breeding, resistance gene screening, and the practical application of apple rootstocks.

## 4. Materials and Methods

### 4.1. Identification of CPP Transcription Factor Family Members in Apple

Whole-genome sequences, annotation files, and protein sequences were retrieved from TAIR (https://www.Arabidopsis.org) (accessed on 21 October 2024) and the Apple Genome and Epigenome Database (https://iris.angers.inra.fr/gddh13/) (accessed on 18 September 2024). A homozygous apple cultivar (doubled haploid of ‘Golden Delicious’) was used in this study. To identify potential *CPP* candidates, eight previously identified AtCPP protein sequences from PlantTFDB (https://planttfdb.gao-lab.org/download.php) (accessed on 20 October 2024) were aligned against the apple protein database in NCBI (https://www.ncbi.nlm.nih.gov) (accessed on 24 October 2024) using BLASTP, with the screening condition E ≤ 10^−10^. The conserved domains of AtCPP family members were analyzed using Pfam (http://pfam-legacy.xfam.org) (accessed on 22 October 2024), confirming the presence of the PF03638 domain. The Pfam pattern databases were downloaded to screen MdCPPs harboring CXC domains [38].

### 4.2. Phylogenetic Tree Construction, Chromosomal Localization, and Protein Physicochemical Characterization

CPP protein sequences in *Arabidopsis*, rice (*Oryza sativa*), grape (*Vitis vinifera*), pear, and tomato (*Solanum lycopersicum*) were obtained from PlantTFDB. A phylogenetic tree was constructed using the neighbor-joining method in MEGA X [63] with 1000 bootstrap replicates and finally visualized using iTOL (https://itol.embl.de) (accessed on 26 October 2024). Chromosomal localization of *MdCPPs* was mapped and visualized using TBtools. Protein physicochemical properties such as molecular weight, isoelectric point, and hydrophilicity index were analyzed. The subcellular localization predictions were performed via Cell-PLoc 2.0 (http://www.csbio.sjtu.edu.cn/bioinf/Cell-PLoc-2/) (accessed on 26 February 2025) [64].

### 4.3. Multiple Sequence Alignment, Conserved Motifs, and Gene Structure Analysis

Multiple sequence alignment of MdCPP proteins was performed using Snapgene software 7.2.1, with annotations of CXC1 and CXC2 domains as well as the conserved R motif. Twelve conserved motifs were identified using the MEME online website and visualized via TBtools. Moreover, the gene structures of MdCPPs were extracted from apple genome annotation files. Finally, integrated visualizations of phylogenetic trees, motifs, and gene structures were generated for comparative analysis [65].

### 4.4. Collinearity Analysis of CPP Genes

Genome sequences and annotation files of *Arabidopsis*, rice, grape, and tomato were downloaded from Ensembl Plants (http://plants.ensembl.org/index.html) (accessed on 10 October 2024), while the pear genome and annotation files were obtained from GIGAdb (http://gigadb.org/dataset/100083) (accessed on 7 November 2024). Gene replication events of *MdCPPs* were analyzed, followed by inter-species synteny analysis between apple, *Arabidopsis*, rice, grape, tomato, and pear. The Ka/Ks ratios of *MdCPPs* were calculated using the Ka/Ks Calculator in TBtools.

### 4.5. Secondary and Tertiary Structure Prediction

The secondary structures of MdCPPs were predicted using the SOPMA (https://npsa.lyon.inserm.fr/cgibin/npsa_automat.pl?page=/NPSA/npsa_sopma.html) (accessed on 27 November 2024). The tertiary structures were modeled and analyzed via the SWISS-MODEL module on the Expasy platform (https://www.expasy.org) (accessed on 20 October 2024).

### 4.6. Cis-Acting Element and Protein Interaction Network Analysis

The 2000 bp upstream sequences of the ATG initiation codon of the *MdCPPs* were submitted to PlantCare online website (https://bioinformatics.psb.ugent.be/webtools/plantcare/html/) (accessed on 20 October 2024) for *cis*-acting element prediction. Statistical analysis of the identified elements was visualized using the ChiPlot online website (https://www.chiplot.online) (accessed on 20 November 2024). For protein interaction network analysis, homologous CPP proteins in *Arabidopsis thaliana* were identified and used to construct protein–protein interaction networks via STRING (https://cn.string-db.org) (accessed on 16 November 2024). The protein–protein interaction network was generated using STRING with a full interactome setting, medium confidence score threshold (≥0.400), and medium stringency (5% FDR).

### 4.7. Analysis of Expression Patterns in Different Organizations

To investigate the expression patterns of *MdCPPs*, the RNA-Seq data of apple were obtained from AppleMDO [66]. The dataset contains samples from various organizations, including leaves, buds, petals, stems, and fruits. The expression levels were normalized as log2 (FPKM + 1) and visualized using heatmaps and schematic diagrams generated using TBtools.

### 4.8. Plant Material Treatment and qRT-PCR Assay

GL-3, selected from the progenies of *Malus domestica* ‘Royal Gala’ [67,68], was grown on Murashige and Skoog (MS) medium supplemented with 0.2 mg·L^−1^ NAA and 0.5 mg·L^−1^ 6-BA (Ph = 5.8) under greenhouse conditions (24 ± 1 °C, 70 ± 5% relative humidity, 16 h/8 h photoperiod, 300 μmol m^−2^ s^−1^ light intensity). Robust apple seedlings, grown for 28 days, were selected and placed in the above-mentioned liquid nutrient solution without agar powder and cultured for one week, followed by hormone treatment. The control treatment refers to cultivating apple seedlings in nutrient solution, while the SA stress treatment refers to apple seedlings in nutrient solution with 100 μM SA added. Subsequently, the apple seedlings were treated with 100 μM GA_3_, 20 μM JA, or 20 μM IAA in a nutrient solution for the respective hormone treatments. Samples containing three biological replicates were collected at 0, 1, 3, 6, 12, and 24 h post-treatment and then immediately frozen in liquid nitrogen to be stored at −80 °C. The total RNA of the leaf samples (0.1 g per plant) was extracted using the RNA Plant Plus Kit (TIANGEN, Beijing, China), and the cDNA was synthesized using the PrimeScript RT Reagent Kit (TaKaRa, Dalian, China). For cDNA synthesis, 1 ug of total RNA was converted using the PrimeScript RT reagent Kit with gDNA Eraser (TaKaRa, Dalian, China). Three biological replicates of each sample were carried out. Eight MdCPP genes (simple random method) were subjected to qRT-PCR analysis using a LightCycler 96 System (Roche, Mannheim, Germany). The data were analyzed using the 2^−ΔΔCt^ method [69], with 18s (Md18s-F, ACACGGGGAGGTAGTGACAA; Md18s-R, CCTCCAATGGATCCTCGTTA) as the internal reference [70]. GraphPad Prism 7.0 was used to draw bar graphs. The primer sequences are listed in Supplementary Table S2.

## 5. Conclusions

This study identified a total of 10 CPP family members and provided a systematic genome-wide analysis in apple. Based on the identification of homologous gene pairs, we explored the expression patterns of *MdCPPs* across different apple tissues and under hormone treatments. The results revealed that *MdCPPs* exhibited distinct responses to varying durations of phytohormone treatments (SA, GA, JA, IAA) and may respond to hormones to combat pathogens and stress. Thus, these findings lay the groundwork for elucidating the functional roles and regulatory mechanisms of CPP proteins in apple. In future research, we will focus on conducting more functional validation experiments to further analyze and explore the gene functions of *CPPs* in apple.

## Data Availability

The data will be made available upon request. The data supporting the findings of this study are available within the paper and its Supplementary Information files. Should any raw data files be needed, they are available from the corresponding author upon reasonable request.

## Supplementary Materials

The following supporting information can be downloaded at https://www.mdpi.com/article/10.3390/ijms26125528/s1.
