# Supplementary material for: Genome-Wide Characterization and Expression Analysis of the Cysteine-Rich Polycomb-like Protein Gene Family in Response to Hormone Signaling in Apple (Malus domestica)
Source: Int J Mol Sci. 2025 Jun 10;26(12):5528. doi: 10.3390/ijms26125528 (PMC12193436; doi:10.3390/ijms26125528)

Supplementary Material 1: **Table S1:** Protein physicochemical properties and subcellular localization of MdCPPs

Sequence ID	Gene Name	Number of Amino Acid	Molecular Weight	Theoretical pl	Instability Index	Aliphatic Index	Grand Average of Hydropathicity	Subcellular localization
MD02G1043000	MdCPP1	593	62963.60	7.71	50.90	62.11	-0.610	Nucleus
MD04G1221500	MdCPP2	455	49404.47	9.05	71.96	73.54	-0.438	Nucleus
MD08G1116500	MdCPP3	650	72020.68	8.78	55.87	71.65	-0.585	Nucleus
MD08G1145800	MdCPP4	900	97609.08	6.38	62.93	63.08	-0.643	Nucleus
MD12G1237500	MdCPP5	658	71990.08	8.00	67.05	69.35	-0.599	Nucleus
MD13G1197800	MdCPP6	787	85924.43	5.63	70.70	59.54	-0.744	Nucleus
MD15G1096100	MdCPP7	576	63245.07	8.79	59.94	61.22	-0.754	Nucleus
MD15G1121200	MdCPP8	897	97764.05	6.58	62.44	62.30	-0.718	Nucleus
MD15G1181800	MdCPP9	594	63350.13	7.72	52.76	63.00	-0.595	Nucleus
MD16G1197800	MdCPP10	790	86331.15	5.38	66.79	61.01	-0.709	Nucleus

Supplementary Material 2: **Table S2:** The specific primers used in the qRT-PCR analysis for the selected *MdCPPs*

Gene_name	Sequence
18S-F	ACACGGGGAGGTAGTGACAA
18S-R	CCTCCAATGGATCCTCGTTA
MdCPP1-F	ACACATCTTCACGGTTGCCT
MdCPP1-R	TGAGTTTCCGCTTCGCTTCT
MdCPP3-F	CCCCTGTAACTCTTGCCCTG
MdCPP3-R	TTTGGCTTGGGGAGAACACA
MdCPP4-F	GCTGGCAGGAGGACTATGAC
MdCPP4-R	ATGTGGAAGAAGCTGAGGCC
MdCPP6-F	TTGTCAGCTCCACTCGTTCC
MdCPP6-R	GAGGCATTGGGGGAATCGAA
MdCPP7-F	TGTCACCTGGAACTCTTGCC
MdCPP7-R	ATGCTCCCCCGCGTAATAAG
MdCPP8-F	CAGCAGAGAAGCCGACAGAA
MdCPP8-R	CTCCCCCTCGTTACATCAGC
MdCPP9-F	TAGTCAATGCTGCACCCGTT
MdCPP9-R	CTGGGTGATGGAGGAGCTTG
MdCPP10-F	ACGTTCAGCTCACTCGGTTT
MdCPP10-R	GTTCGGCCGCATCTTCATTC

Supplementary Material 3: **Table S3:** Evolutionary Pressure Analysis of *MdCPPs*. The Ka, Ks, and Ka/Ks values of 6 pairs of *MdCPPs* homologous gene pairs are shown in the table

Homologous gene pairs	Ka	Ks	Ka/Ks
MdCPP1/MdCPP9	0.037	0.17	0.218
MdCPP2/MdCPP6	0.427	1.873	0.228
MdCPP3/MdCPP7	0.059	0.173	0.341
MdCPP4/MdCPP8	0.062	0.149	0.416
MdCPP5/MdCPP6	0.434	1.91	0.227
MdCPP6/MdCPP10	0.046	0.194	0.237

Supplementary Material 4: **Figure S1:** The CXC conserved domain of MdCPPs

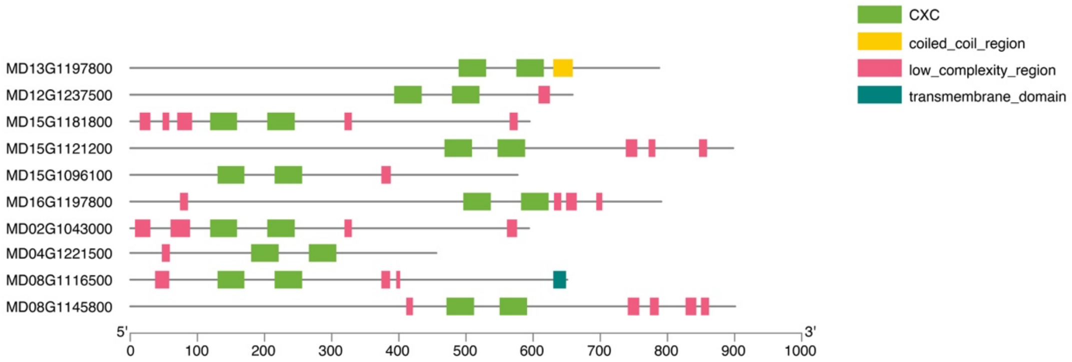

Supplement: Supplementary file 1 [file ijms-26-05528-s001.zip › ijms-3632401-supplementary.pdf]
